# Survey of the initial management of celiac disease antibody tests by ordering physicians

**DOI:** 10.1186/s12887-019-1621-5

**Published:** 2019-07-19

**Authors:** Kathryn Potter, Lawrence de Koning, J. Decker Butzner, Dominica Gidrewicz

**Affiliations:** 1 Department of Pediatrics, Cumming School of Medicine, University of Calgary, Calgary, Canada; 2 Department of Pathology and Laboratory Medicine, Cumming School of Medicine, University of Calgary, Calgary, Canada; 3 Calgary Laboratory Services Calgary, Calgary, Alberta Canada; 4 Division of Pediatric Gastroenterology, Alberta Children’s Hospital, 2888 Shaganappi Trail, Calgary, AB T3B 6A8 Canada

**Keywords:** Celiac disease, Tissue transglutaminase, Pediatric, Diagnosis

## Abstract

**Background:**

Appropriate interpretation of a positive celiac antibody test by an ordering physician is important in order to institute proper management. We evaluated why children with an initial positive celiac serology were not referred for diagnostic biopsy or followed with serial testing by the ordering physician.

**Methods:**

Consecutive celiac serologies in all patients less than 18 years of age were evaluated over 3.5 years and 775 children with a positive tissue transglutaminase antibody (TTG) were identified. If no management of a positive TTG could be identified, a survey was sent to the ordering physician. Responses were categorized as appropriate or inappropriate management.

**Results:**

Of the 775 patients with a positive TTG, 193 (24.9%, 95% CI 21.9–28.1%) received no follow-up management. We contacted 173 ordering physicians and 120 (69%) responded. Of the 120 responses, 55 patients (45.8%, 95% CI 36.8–55.1%) were managed appropriately and 46 (38.3%, 95% CI 29.7–47.7%) were considered to be inappropriately managed when no repeat TTG was obtained within 18 months. Reasons for inappropriate management included: screen considered to be false positive (44.7%), patient was not experiencing symptoms of celiac disease (31.6%), symptoms had resolved (15.8%), results were not indicative of celiac disease (26.3%) and patients started a gluten-free diet with no evaluation of response (15.8%). In 19 patients the TTG was not acted upon for technical reasons.

**Conclusions:**

Positive TTGs require appropriate interventions. These include: subspecialist referral for further evaluation and/or repeat testing to evaluate: 1) treatment response or 2) patients with minimal or no symptoms.

**Electronic supplementary material:**

The online version of this article (10.1186/s12887-019-1621-5) contains supplementary material, which is available to authorized users.

## Background

Celiac disease is an autoimmune enteropathy triggered by ingested gluten in genetically susceptible individuals, and affects approximately 1% of the population [1, 2]. Timely and accurate diagnosis of celiac disease is limited because physicians do not recognize atypical presentations, including chronic abdominal pain or chronic constipation [3, 4]. In addition approximately 60% of children present with extra-intestinal manifestations of celiac disease including fatigue, headaches, growth failure, arthralgias, and iron deficiency anemia [3, 5, 6]. Many physicians are not aware of these atypical presentations of celiac disease that result in delays in diagnosis [7, 8]. Two-thirds of patients consult at least two physicians prior to the diagnosis [8]. Delayed diagnosis and persistent intestinal inflammation lead to complications that include persistent gastrointestinal symptoms, pubertal delay, osteoporosis, autoimmune disorders, and increased risk of non-Hodgkin lymphoma [9–13]. An economic evaluation demonstrated that in the year pre-diagnosis, persistent health concerns were investigated at an estimated cost of $8500 US per capita [14]. Delayed diagnosis taxes both the individual and the health care system.

Celiac disease antibody screening with the tissue transglutaminase (TTG) antibody test identifies individuals who require further testing to diagnose celiac disease or alterations in management of those with known celiac disease. Management decisions following a positive celiac screen are influenced by the degree of elevation of the TTG and severity of patient symptoms. In addition, the TTG may be falsely positive in individuals with minimal or no symptoms such as first-degree relatives of patients with celiac disease or individuals who have medical conditions associated with celiac disease [4]. Unfortunately other autoimmune diseases and intercurrent infections can cause false positive TTG results and must be considered when managing patients with minimal symptoms [15]. Thus, many factors influence how celiac serology should be interpreted. “Choosing Wisely” is an initiative to help clinicians to appropriately manage abnormal test results and reduce unnecessary testing [16]. Physicians must understand when a test should be ordered and how to interpret the result. By identifying general errors in management, physicians can improve patient care and reduce health care costs.

Appropriate management of children with possible celiac disease begins with correct identification of those children that require further testing and/or subspecialty evaluation. We hypothesized that a proportion of children who do not undergo an intestinal biopsy did not receive further evaluation of an initial positive celiac screen and/or started a gluten-free diet without ongoing monitoring of their celiac serology. Thus, the aim of our study was to evaluate the management of an initial positive celiac screening test by the ordering physician in order to understand how physicians manage celiac serology test results. The data were used to develop educational strategies that address these issues in order to decrease the time to diagnosis and improve quality of care for children with celiac disease.

## Methods

Consecutive patients (< 18 years of age) with celiac serology were identified in the Calgary Laboratory Services (CLS) database over 3.5 years from July 2008 to December 2011. All patients had a serum IgA and IgA-tissue transglutaminase (TTG) performed (Euroimmune, Germany). If the TTG was positive (> 20 kU/L), an IgA anti-endomysial antibody (EMA) test was measured (IMMCO Diagnostics, NY). Intestinal biopsy results were obtained from the Alberta Children’s Hospital pathology database. This hospital is the only pediatric gastroenterology referral center in southern Alberta, Canada, that provides service to a large pediatric population of over 460,000 children. It is staffed by nine pediatric gastroenterologists.

The electronic medical record, clinic charts and small intestinal pathology reports were obtained on all patients with a positive TTG. Those patients with no record of intestinal biopsy or consultation with a pediatric gastroenterologist were identified for the survey study. A questionnaire (Additional file 1: Figure S1) was mailed to the ordering physician to evaluate the management of these TTG-positive patients.

Patients were classified into three groups: high TTG (≥ 10 x the upper limit of normal, ULN, ≥ 200 kU/L), moderately elevated TTG (3–10 x ULN, 60–199 kU/L), and low TTG (< 3 x ULN, 20–59 kU/L). These cut-offs corresponded to those set out in the ESPGHAN guidelines on the diagnosis of celiac disease in children [4, 17]. Physician management in this study occurred prior to the publication of these guidelines. Management was considered appropriate if patients with a TTG ≥ 3 x ULN were referred for evaluation by a gastroenterologist. Patients with minimal or no symptoms and a TTG of < 3 x ULN could be referred to a pediatric gastroenterologist or followed with subsequent celiac antibody levels to evaluate if they decreased [4, 17]. Patients with a TTG < 3 x ULN and negative EMA have only a 13% positive predictive value of biopsy proven celiac disease [17]. Inappropriate management was assigned to patients with a TTG ≥ 3 x ULN (≥ 60 kU/L) who were not referred and did not have repeat serology within 18 months of the first celiac screen. Patients with a TTG ≥ 10 x ULN and positive EMA (who may qualify for a non-biopsy diagnosis under ESPGHAN criteria) were assigned to the inappropriate management category if no repeat TTG was performed.

Where appropriate, mean, median, significant difference between proportions and 95% confidence intervals (CIs) were calculated. The study was approved by the University of Calgary Medical Ethics Conjoint Health Research Ethics Board.

## Results

Of the 17,505 children who had celiac serology performed, 775 patients had a positive TTG [17]. Of these, 543 (70%; 95% CI 66.7–73.2%) were referred to the pediatric gastroenterology service at the Alberta Children’s Hospital, 39 (5.0%; 95% CI 3.6–6.9%) with known celiac disease had repeat serology, and 193 patients (24.9%; 95% CI 21.9–28.1%) with a positive TTG were not referred for pediatric gastroenterology evaluation. Of the 193, the ordering physician was not identified in 20 patients. Thus 173 surveys were distributed and 120 were completed and returned. The study response rate was 69.4%, composed of family practitioners (57.8%), pediatricians/pediatric subspecialists (38.5%) and internal medicine subspecialists (3.6%). Of the pediatricians, 59.3% were community pediatricians, 12.5% hospitalists, and 28.2% pediatric subspecialists. Of the 775 patients with a positive TTG, we could not evaluate management in 73 (9.4%, Table 1).

**Table 1:** Summary of patients in whom follow-up management was evaluated (*n* = 193)

	Appropriate (*n* = 55)	Inappropriate (*n* = 46)	Technical (n = 19)	Nonresponders (*n* = 73)
Mean age at initial TTG (yrs)	9.9 +/− 5.2	9.1 +/− 4.8	9.9 +/− 5.0	9.2 +/− 5.25
Female, n (%)	27 (49.1)	23 (50.0)	13 (68.4)	49 (67.1)
EMA positive, n (%)	10 (16.7)	7 (15.2)	8 (42)	19 (26)
TTG, n (%)				
≥ 10 x ULN	4 (7.3)	5 (10.9)	6 (31.6)	17 (23.3)
3–10 x ULN	15 (27.3)	10 (21.7)	2 (10.5)	14 (19.2)
1–3 x ULN	36 (65.4)	31 (67.4)	11 (57.9)	42 (57.5)

*EMA* anti-endomysial antibody

*TTG* tissue transglutaminase antibody

*ULN* upper limit of normal

The only statistical difference was between the appropriate & inappropriately managed patients with a TTG ≥ 10 x ULN (9/101) versus the nonresponders with a TTG ≥ 10 x ULN (17/73) (*p* < 0.05)

Of the 120 patients with completed questionnaires, 55 (45.8%, 95% CI 36.8–55.1%) patients were considered to be managed appropriately. This included either a repeat TTG within 18 months of the previously elevated TTG and/or referral for subspecialty evaluation for further management (Table 1, Additional file 2: Figure S2). All of the nine patients with a TTG > 3 x ULN and positive EMA were appropriately managed within four months (Additional file 2: Figure S2). Of those with a moderately elevated TTG (3–10 x ULN), 8/15 were determined likely a false positive and the TTG was repeated a median 3.5 months later (range 2.5 to 15 months). The TTG returned to normal in six patients or was only 1.5 x ULN in two. Of the 36 with a low TTG (< 3 x ULN), four had known celiac disease, six were referred for subspecialty evaluation and 26 patients had a repeat TTG a median of 6 months later (range 1–18 months). In 18 patients, the TTG had normalized upon repeat testing.

Of the 120 patients, 46 (38.3%, 95% CI 29.7–47.7%) were managed inappropriately including 18 children who had a repeat TTG at least 3.5 years after the initial TTG and 28 children with no repeat TTG (Table 1). All five of the patients with a TTG ≥ 10 x ULN started a GFD without appropriate follow-up testing to assess response to a GFD (four patients had no repeat TTG, one had a repeat TTG over 6 years later). Of the 10 patients with a moderately elevated TTG (3–10 x ULN), only five had a repeat TTG (median 38 months later, range 21–50 months), while five had no repeat TTG and the ordering physician did not feel the test was indicative of celiac disease based on the patient’s symptoms. Two-thirds of inappropriately managed patients (31/46) had a low TTG (< 3 x ULN). Only 12 had a repeat TTG (median 37 months, range 25–83 months), of which four patients had an increased TTG upon repeat testing. Nine patients who refused GI evaluation did not have a repeat TTG.

As physicians may act more cautiously with younger patients, we evaluated if the patient’s age at initial TTG influenced a physician’s follow-up management. We characterized the appropriately and inappropriately managed patients into two groups: under six years of age (*n* = 26) and greater than six years (*n* = 75). Of the children less than six, 12/26 (46.1%; 95% CI 28.7–64.5%) were inappropriately managed, as compared to 34/75 (45.3%; 95% CI 34.6–56.6%) of those over six, thus showing a similar proportion of inappropriately managed patients in both groups.

The TTG was not acted upon for technical reasons in 19 patients (15.8%, 95% CI 10.0–23.9%) (Table 1). The TTG was classified as a technical error when the ordering physician: was unable to contact the patient or the patient moved away (6), did not order the test (10), did not receive the results (1), or patient records were no longer available (2).

The most common reasons for not referring for gastrointestinal evaluation included: the screen was considered a false positive (51.2%), the patient was not experiencing symptoms of celiac disease (39.5%), or the results did not appear indicative of celiac disease (30.2%) (Table 2).

**Table 2:** Reason(s) for not referring for gastrointestinal evaluation (n = respondents) n (%)

	Appropriate (*n* = 33)	Inappropriate (*n* = 38)
Low antibody titre likely a false positive	17 (51.5)	17 (44.7)
Patient was not experiencing symptoms of CD	13 (39.4)	12 (31.6)
Results did not appear to be indicative of CD	10 (30.3)	10 (26.3)
Patient unwilling to undergo intestinal biopsy	5 (15.2)	8 (21.0)
Symptoms had resolved	3 (9.1)	6 (15.8)
Patient already on a gluten-free diet	2 (6.1)	6 (15.8)
False positive due to a known medical condition	8 (24.2)	3 (7.9)

Sixty-five physicians listed sources of information for the diagnosis and management of celiac disease. The majority of respondents derive their information from formal continuing medical education, including medical journals (34%), conferences (25%), Internet materials (22%) and guidelines (8%). Other sources included expert opinion (46%), residency training (18%) and other (9%).

## Discussion

This observational survey evaluated management decisions of celiac disease antibody tests by ordering physicians. As the first point of contact for patients, the rationale behind proper physician management of a positive celiac screen has important implications for the patients. Of the patients with a positive celiac antibody test who were not referred to a gastroenterologist, 38% were managed inappropriately. The frequency of inappropriate management of patients could rise to as high as 71% if all survey “technical no contact” and “non-responder” individuals are included in that group.

We identified multiple reasons why physicians inappropriately managed celiac disease antibody tests. Of the patients with a low titer TTG (< 3 x ULN) and inappropriate management, physicians indicated that the initial screen was indicative of a false positive such as due to lack of symptoms or symptoms had resolved. However, in order to properly diagnose a false positive test in these situations, a patient must have a repeat TTG that normalizes while consuming gluten. A low positive TTG may indicate early celiac disease. In fact, 19% of the patients who had a repeat TTG over three years later, displayed an increased TTG and experienced a delayed diagnosis of celiac disease. Additional erroneous assumptions that led to inappropriate management included: no referral because symptoms resolved, starting on a GFD with no follow-up testing to evaluate response to a gluten free diet, and patient refusal for further evaluation. Families may lack the understanding to differentiate between a screening test result with the potential for a false positive result and a gold-standard diagnostic test, such as an intestinal biopsy. In addition, children with minimal or no symptoms and a weakly positive antibody test may develop celiac disease with higher antibody levels over time [4]. Repeat antibody testing is required to evaluate these clinical scenarios.

For the appropriate interpretation and management of a positive screening test, one must stratify patients into low-risk patients who are unlikely to have celiac disease, and identify high-risk patients who require an intestinal biopsy to confirm the diagnosis and to evaluate response to a GFD [4, 9, 17]. Although there is growing awareness of celiac disease, adults without gastrointestinal symptoms have a mean delay in diagnosis of 42 months [7]. Long delays in diagnosis contribute to a negative impact on patient quality of life and increase health utilization and costs [14]. This study reflects the goals of the “Choosing Wisely” campaign that seeks to encourage physicians to make effective choices to ensure high quality care [16]. In order to improve how physicians choose to order, interpret, and follow celiac serology tests, one must first identify incorrect assumptions in laboratory test interpretation and subsequently develop educational tools to improve primary care physician management decisions.

Given the large proportion of asymptomatic patients with a positive TTG on a gluten containing diet who are at risk of celiac disease complications, a positive celiac screen needs to be serially monitored. If the initial TTG is positive but low (< 3 x ULN) with a negative EMA, then a false-positive result is likely [4, 17]. In the absence of any signs or symptoms, the ordering physician may follow the child on a gluten-containing diet and serological testing repeated every 6–12 months until the antibody levels normalize or increase to a level where biopsy is indicated. If the EMA is positive, then the likelihood for celiac disease increases because of the high specificity of EMA, and such patients, even at low titer TTGs, should be referred for specialist evaluation and endoscopy [4, 17].

The clinical presentation of a patient influences a physician’s interpretation of the initial TTG. Though a physician may choose to act more cautiously in younger children, our study did not show that children over six years of age were more likely inappropriately managed compared to younger children. Atypical presentations, however, are increasingly common in children [4–6], such that close to 60% of children may be asymptomatic or present atypically [5]. A common physician error was the assumption that a patient with a positive screening test was less likely to have celiac disease if the child lacked classic symptoms. A large study identified seven new cases of celiac disease presenting atypically in children for each new case of celiac disease that presented with the classical symptoms of celiac disease [18]. Therefore, current pediatric and adult guidelines recommend testing for celiac disease in a large variety of ailments, including nonspecific gastrointestinal symptoms such as chronic abdominal pain or IBS, and non-GI (atypical) symptoms such as nutritional anemia, chronic fatigue, short stature, delayed puberty, dental enamel defects, elevated liver transaminases levels and dermatitis herpetiformis [4, 11].

Furthermore, 15% of patients without appropriate follow-up serial TTG started a gluten-free diet prior to diagnosis against medical advice. It is important for medical practitioners to emphasize that a confirmed diagnosis is essential to justify starting a gluten-free diet that must be followed for life. This diet is expensive, complicated and difficult to manage, and therefore patients with newly diagnosed celiac disease should be strongly encouraged to meet with a dietitian skilled in the gluten-free diet to prevent dietary contamination and assist with long-term adherence [4, 9].

This study provides valuable information as it includes a comprehensive number of physicians and their management of elevated celiac screen. The study has limitations including the retrospective data collection and recall bias as the surveys were mailed out up to 4 years after the original TTG was ordered. We were unable to verify that all TTG screens were done on patients who consumed gluten. In those who underwent repeat serology, we could not assess dietary compliance. A response rate of 68% is acceptable but not complete.

## Conclusions

This observational study demonstrates common errors in celiac serology interpretation by ordering physicians that include failure to: follow up low titres, recognize that celiac disease may occur in patients without classic celiac symptoms, and evaluate TTG serologies of patients who decline duodenal biopsy, including those who start a gluten-free diet. Physicians need to be educated about the risks of undetected celiac disease in asymptomatic or mildly symptomatic patients and the higher incidence of celiac disease in autoimmune conditions that can cause false positive screening tests. Positive TTGs, even < 3 x ULN, need follow up within a year, and if titres increase a consultation for further evaluation. TTGs ≥ 3 x ULN should be referred for consultation for intestinal  biopsy and those diagnosed with celiac disease need periodic repeat TTGs to evaluate response to a gluten-free diet. Improvement in the management of positive celiac disease screening tests will lead to early disease identification, minimize unnecessary testing for other conditions, improve patient’s quality of life and reduce healthcare costs.

## Additional file

Additional file 1: **Figure S1.** Study questionnaire. (PDF 124 kb)

Additional file 2: **Figure S2.** Study flow diagram. (PPTX 32 kb)

## Data Availability

The datasets used and/or analysed during the current study are available from the corresponding author on reasonable request.

## Abbreviations

CI: Confidence Interval
EMA: Anti-endomysial antibody
GFD: Gluten-free diet
TTG: Tissue transglutaminase
ULN: Upper limit of normal
