# Supplementary material for: Survey of the initial management of celiac disease antibody tests by ordering physicians
Source: BMC Pediatr. 2019 Jul 19;19:243. doi: 10.1186/s12887-019-1621-5 (PMC6639898; doi:10.1186/s12887-019-1621-5)
Supplement: Supplementary file 1 — Figure S1. Study questionnaire. (PDF 124 kb) [file 12887_2019_1621_MOESM1_ESM.pdf]

CELIAC DISEASE SEROLOGICAL TESTING QUESTIONNAIRE

1 Please indicate the appropriate response

	This is not my patient
	This is a patient who was formerly in my care
	This is a patient who remains in my care (Please indicate date of last visit)

2. If this is a former or current patient, please indicate the appropriate response regarding celiac screening tests ordered

	I ordered the celiac screen and followed up on results.
	This test was not ordered by me
	The incorrect test was done by the laboratory
	The patient records are no longer available
	The results were not received by me
	Other

3a. Was a biopsy performed as a follow up to this celiac screen? [J Yes or [J No

If yes, please indicate date, location of biopsy, results of test and pathology reference number (if available)

3b. If you answered NO to question 3a, please indicate clinical rationale by choosing each option that applies to your patient (multiple choices may be made)

	I was unable to contact the patient	
	Test results indicated a low antibody titre that is likely a false positive	
	Test results were likely a false positive due to a known medical condition (eg. type 1 diabetes, inflammatory bowel disease, or liver disease) Please indicate condition	
	Test results did not appear to be indicative of celiac disease	
	Patient was not experiencing symptoms of celiac disease	
	Symptoms had resolved	
	Patient was unwilling to undergo intestinal biopsy	
	Patient was unable to undergo intestinal biopsy for medical reasons (Please specify	
	Patient had previous intestinal biopsy and this was a follow up test. Please provide further details below	
		Patient has biopsy-proven celiac disease, serology done to demonstrate improvement on a gluten free diet.
		Patient has biopsy-proven celiac disease, serology was to assess compliance with a gluten-free diet
		Previous biopsy was not diagnostic of celiac disease and serology was done to evaluate for higher titres to determine if a 2"d biopsy should be done.
		Other
	Patient had already started on a gluten-free diet. Please provide further details below.	
		Prolonged wait for consultation with gastroenterologist
		Patient's history or family history was strongly suggestive of celiac disease
		Patient began a gluten free diet despite physician's recommendation for a biopsy
		Other
	Patient is currently awaiting an intestinal biopsy	
	Other	

4. What sources did you use to learn about the use of serology in the diagnosis and management of celiac disease? (Mark all that apply.)

	Medical journals		Alberta celiac disease guidelines
	Continuing medical education		Expert opinion
	Conferences		Web-based sources
	Residency training experience		Other
