# Supplementary material for: Survey of the initial management of celiac disease antibody tests by ordering physicians
Source: BMC Pediatr. 2019 Jul 19;19:243. doi: 10.1186/s12887-019-1621-5 (PMC6639898; doi:10.1186/s12887-019-1621-5)
Supplement: Supplementary file 2 — Figure S2. Study flow diagram. (PPTX 32 kb) [file 12887_2019_1621_MOESM2_ESM.pptx]

## Slide 1
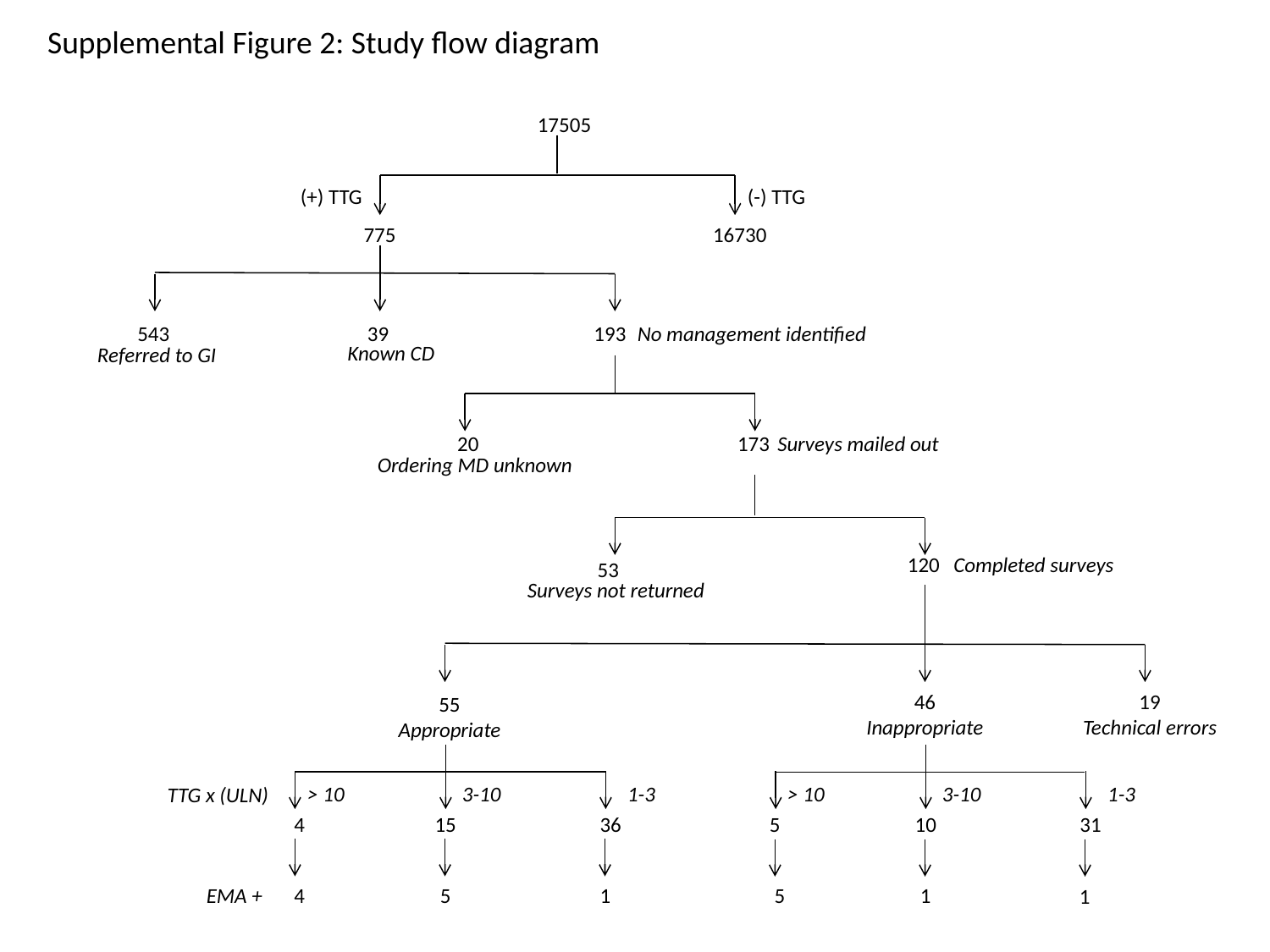

Supplemental Figure 2: Study flow diagram
17505
(+) TTG
(-) TTG
775
16730
543
39
193
No management identified
Known CD
Referred to GI
20
173
Surveys mailed out
Ordering MD unknown
120
Completed surveys
53
Surveys not returned
46
Inappropriate
19
Technical errors
55
Appropriate
> 10
3-10
1-3
> 10
3-10
1-3
TTG x (ULN)
4
15
36
5
10
31
1
EMA +
4
5
5
1
1
